# Artificial Intelligence and Machine Learning Applied at the Point of Care

**DOI:** 10.3389/fphar.2020.00759

**Published:** 2020-06-18

**Authors:** Zuzanna Angehrn, Liina Haldna, Anthe S. Zandvliet, Eva Gil Berglund, Joost Zeeuw, Billy Amzal, S. Y. Amy Cheung, Thomas M. Polasek, Marc Pfister, Thomas Kerbusch, Niedre M. Heckman

**Affiliations:** ^1^Certara, Princeton, NJ, United States; ^2^PacMed, Amsterdam, Netherlands; ^3^Department of Clinical Pharmacology, Royal Adelaide Hospital, Adelaide, SA, Australia; ^4^Centre for Medicines Use and Safety, Monash University, Melbourne, VIC, Australia; ^5^Department of Pharmacology and Pharmacometrics, Children's University Hospital Basel, Basel, Switzerland

**Keywords:** software as a medical device, Artificial Intelligence and Machine Learning in medical practice, chronic disease management, clinical decision support tools, precision dosing, real-world evidence, model-informed precision dosing

## Abstract

**Introduction:**

The increasing availability of healthcare data and rapid development of big data analytic methods has opened new avenues for use of Artificial Intelligence (AI)- and Machine Learning (ML)-based technology in medical practice. However, applications at the point of care are still scarce.

**Objective:**

Review and discuss case studies to understand current capabilities for applying AI/ML in the healthcare setting, and regulatory requirements in the US, Europe and China.

**Methods:**

A targeted narrative literature review of AI/ML based digital tools was performed. Scientific publications (identified in PubMed) and grey literature (identified on the websites of regulatory agencies) were reviewed and analyzed.

**Results:**

From the regulatory perspective, AI/ML-based solutions can be considered medical devices (i.e., Software as Medical Device, SaMD). A case series of SaMD is presented. First, tools for monitoring and remote management of chronic diseases are presented. Second, imaging applications for diagnostic support are discussed. Finally, clinical decision support tools to facilitate the choice of treatment and precision dosing are reviewed. While tested and validated algorithms for precision dosing exist, their implementation at the point of care is limited, and their regulatory and commercialization pathway is not clear. Regulatory requirements depend on the level of risk associated with the use of the device in medical practice, and can be classified into administrative (manufacturing and quality control), software-related (design, specification, hazard analysis, architecture, traceability, software risk analysis, cybersecurity, etc.), clinical evidence (including patient perspectives in some cases), non-clinical evidence (dosing validation and biocompatibility/toxicology) and other, such as e.g. benefit-to-risk determination, risk assessment and mitigation. There generally is an alignment between the US and Europe. China additionally requires that the clinical evidence is applicable to the Chinese population and recommends that a third-party central laboratory evaluates the clinical trial results.

**Conclusions:**

The number of promising AI/ML-based technologies is increasing, but few have been implemented widely at the point of care. The need for external validation, implementation logistics, and data exchange and privacy remain the main obstacles.

## Introduction

The healthcare industry is changing rapidly due to increasing demand and new technological developments. Medicinal and therapeutic options are becoming more complex and personalized, causing regulatory and health technology assessment, reimbursement, and therefore, healthcare accessibility to be challenge in most countries.

In today's medical practice, patient support tools and Model-Informed Precision Dosing (MIPD) offer practitioners an additional arsenal for overseeing an individual's best treatment options. Digital disease management platforms are aiming to improve patient outcomes, leveraging the best medical care options, and augmenting physicians' knowledge processing, while reducing overall healthcare costs. As such, there are numerous opportunities for Artificial Intelligence (AI)- and Machine Learning (ML)-based solutions to enhance and personalize medical practice.

While there has been an increasing trend in the digital transformation of healthcare, the initial exuberance is now maturing. Large pharmaceutical companies are heavily investing in the digital transformation and are creating digital health departments. They now list Google and IBM as their main future competitors. The equity markets have grown from an investment of $9.5 billion in digital health applications in 2018 to an estimated $20 billion today (Zion, 2019). The increasing availability of healthcare data and rapid development of big data analytic methods opened new avenues for use of AI and ML at the point of care. The Food and Drug Administration (FDA) is on board. FDA has recently issued a statement on new steps to advance digital health policies (FDA, 2019f) that encourage innovation and enable efficient and modern regulatory oversight and proposed a regulatory framework for AI- and ML-based software as medical devices (FDA, 2018e).

## Introduction to Machine Learning

McCarthy defined AI as “the science and engineering of making intelligent machines, especially intelligent computer programs” (McCarthy, 2007). ML is a subset of AI in which the algorithm allows computer programs to improve without additional programming. Instead, the software learns through inferential experience (Mitchell, 1997; Expert System, 2017).

Typical tasks of ML algorithms include classification, prediction, pattern recognition, and clustering and feature identification. In the healthcare arena, this includes for example classifying patient profiles (e.g. responders vs. non-responders to a treatment), defining proxies for diagnosis or prognosis, predicting long term outcomes or interpreting medical images. Unsupervised ML recognizes patterns in data without pre-specified structure. Conversely, supervised learning algorithms are trained on labeled structured data.

Another categorization of ML methods differentiates classical learning algorithms from Deep Learning:

1. *Classical ML algorithms:* they include the most common ML algorithms such as regression models, Support Vector Machines as well as tree-based methods. Common types of tree-based models are Decision Trees, Random forest, and Gradient Boosting. These models split the set of data points in subsequent steps based on the values of selected features, aiming to identify homogeneous subsets of data points from the overall heterogeneous set. The goal is to identify and cluster subsets that are highly comparable in the features describing them, as well as their outcome.
2. *Deep Learning (DL) algorithms:* also simply called Neural Networks, deep learning models create complex combinations of the original features in subsequent combinations layers. The more layers, the ‘deeper' the model, hence the name. The major advantage of this type of models is to identify higher level features that have far more predictive value than the original features. Common applications include medical image analysis and natural language processing applied to large text mining and big medical database curation. **Box 1** presents the process involved in developing a machine learning model.

Box 1 **Process of Developing a Machine Learning Model.**

Developing a ML algorithm is a step-wise process that allows to classify subjects or predict outcomes from a set of raw data:

1. *Data Preprocessing*: in the preprocessing phase the raw data is analyzed and cleaned. Corrupt data points are identified and removed, data points (e.g. individual cases where an outcome is to be predicted) and relevant variables are selected, and the data (often from multiple data sources) is transformed into a single dataset which has a tabular format.
2. *Feature Engineering:* the raw variables in the data are transformed into features. This process is characterized by manipulating and combining raw features to increase their predictive value (e.g. combining weight and length of a patient to produce the more relevant BMI). Categorical variables are converted into dummy features, and numerical features can be normalized or converted into categories (e.g. transforming the numerical age to 5 age-categories)
3. *Modeling:* after transforming the data into a format that can be analyzed by a ML model, an initial model is trained (the different types of models are presented in the section below). The predictive performance of this standard, non-optimized model is usually used as a benchmark to assess the added value of different optimization techniques.
4. *Evaluation*: the performance of the model is evaluated, and the predictive value of the different features is analyzed. Based on these evaluations, developers go back to the feature engineering to manipulate the features and increase their predictive value in a hypothesis-based approach from the insights of the modeling.
5. *Model optimization*: in the optimization phase a number of different types of ML models are developed and evaluated. Based on the type of data/problem, amount of data, and experience of the developers make some models more suitable than others. The final selection for a type of model is, however, predominantly based on trial-and-error and learning which type of model provides the best performance for the specific problem. After the most suitable type of model is selected, the hyper-parameters (internal settings) of the model are optimized by testing different configurations.

This article presents a selection of typical clinical applications of ML. First, tools for monitoring and remote management of chronic diseases are presented. Second, imaging applications for diagnostic support are discussed. Third, CDSS (Clinical decision support software) to facilitate precision dosing are reviewed. In addition, evidence scope and regulatory requirements in the United States (US), Europe, and China for applying AI/ML-based tools and devices in the healthcare setting are discussed.

## Materials and Methods

A case series that is backed up by non-systematic searches in academic databases and internet search engines is presented. To identify regulatory requirements for introducing ML-based solutions to the market, a targeted search and a narrative review were performed on the websites of regulatory agencies in the US, Europe, and China.

## Results

Four use cases representing a spectrum of clinical situations were selected and described below.

### Case 1: Home Monitoring and Remote Management

Wearable technologies to detect motion, heart rate, and other functional or physiological variables are widely available by many providers (such as Fitbit^®^, Apple Watch^®^, Garmin^®^, and Samsung Galaxy^®^, among others). Devices such as fitness devices and smart watches are commonplace, with one in six consumers in the US (Statista, 2019). Given the increasing needs and value of real-world and real-time healthcare data, there has been growing interest in such technologies for monitoring chronic diseases as well as for earlier detection or alerts of clinical events such as atrial fibrillation or stroke. Wearables for diabetes management will most likely be one high-impact use case in the short term. As wearable insulin measuring devices are already widely available, the next step could be the automation of insulin administration, with minimal human intervention.

For example, Tandem Diabetes Care has gained approval in the US and Canada for their t:slim X2™ insulin pump with Basal-IQ™ technology (FDA, 2018f; Tandem, 2019b). Basal-IQ technology, a predictive low-glucose suspend algorithm, utilizes sensor values from an integrated Dexcom G6 continuous glucose monitor (CGM) to help reduce the frequency and duration of low-glucose events (hypoglycemia). Dexcom G6™, which is also an independently functioning constant glucose monitor, is provided by Dexcom Inc. (Dexcom, 2019). Using Dexcom G6™ CGM values, the Basal-IQ™ feature predicts the glucose level 30 min ahead and suspends insulin when the glucose level is predicted to drop below 4.4 mmol/L (80 mg/dl) or if the glucose level is currently below 3.9 mmol/L (70 mg/dl). The system resumes insulin administration once the sensor detects that the glucose values start to rise. Integrated with the Dexcom G6 CGM, the Basal-IQ is based on simple linear regression algorithm that predicts glucose levels 30 min ahead based on three of the last four consecutive CGM readings. The feature works with no finger sticks blood draw required (if glucose alerts and CGM readings do not match symptoms or expectations, a blood glucose meter should be used to make treatment decisions). t:slim X2™ can suspend insulin for up to 2 h within a 2.5-hour rolling window (Tandem, 2019a).

As part of the approval process in the US and Canada, Tandem Diabetes Care has also conducted several studies on the technology (Rajani et al., 2015; Brown et al., 2019). Firstly, a six-week randomized pivotal crossover study comparing two 3-week periods of at-home insulin pump use, one period using the t:slim X2™ pump with Basal-IQ™ technology, and another period using a CGM-integrated t:slim X2™ pump without automated insulin suspension. The use of Basal-IQ™ technology reduced the number of sensor glucose readings below 3.9 mmol/L (70 mg/dl) by 31% compared to the control period using a standard CGM-integrated t:slim X2™ pump without automated insulin suspension. Marked reduction of sensor time below 3.9 mmol/L (70 mg/dl) was accomplished without any increase in the frequency of hyperglycemia, and patients described the system as simple to learn and use (Forlenza et al., 2018). Secondly, data published on real-world use of Basal-IQ technology has demonstrated even greater reductions in time spent below 3.9 mmol/L (70 mg/dl) than those seen in the pivotal trial (Muller et al., 2019).

### Case 2: Feature Detection and Diagnosis Support

Another major area of AI/ML applications is AI-based diagnosis support. Such technologies may process any clinical and nonclinical data as well as images, such as X-ray, computer tomography (CT), magnetic resonance imaging (MRI), ophthalmic images, etc.

The volume of medical images requiring interpretation have been increasing systematically in the recent decades, reaching for example 1 billion radiographic examinations annually (Waite et al., 2017) and exerting a pressure on the healthcare systems to secure an increasing number of radiologists. Even when radiologists are available and extensively trained, the interpretation of medical images is still prone to biases and errors whose rates have not diminished for decades (Waite et al., 2019); these commonly lead to misdiagnosis (Goergen et al., 2015). ML holds the promise of breaking this impasse by efficient and accurate analysis of large volumes of medical imaging data, hence with substantial physician's time saving. A clear advantage of ML methods is that they can be directly applied to image, without the need to extract numeric variables, and feature extraction and classification can happen simultaneously.

One of the most sophisticated AI-based diagnosis support is CT-Flow (HeartFlow^®^) software for non-invasive diagnosis and management of coronary artery disease (CAD) based on a coronary computerized tomography angiography (CCTA) scans and patient characteristics (HeartFlow, 2019). HeartFlow's coronary vascular physiologic simulation software was cleared by the FDA *via* the De Novo (DEN130045) regulatory pathway on November 26, 2014 (classification product code PJA; regulation number 21 Code of Federal Regulations (CFR) 870.1415, class II, 510(k) regulatory pathway). Traditionally, coronary angiography is used to evaluate the flow of blood in the blood vessels, this approach generates risk of adverse events, including death. HeartFlow^®^ provides a safe alternative—if signs of CAD are present in a CCTA, the images are sent to a cloud-based HeartFlow^®^ server for post-processing by a set of algorithms, supported by trained analysts. ML and DL algorithms are then used to build a physiological model of a patient's heart based on the CT scan and computational fluid dynamics. These are applied to estimate blood pressure, velocity, and flow, and thereby assess the blood flow in the coronary arteries. In the final step a personalized, color-coded 3-dimensional model of a patient's coronary arteries is constructed, indicating functional information about each blockage to a physician. The model is stored in the system and fed into the DL algorithm, contributing to its improved performance in future applications. Schematic outline of this analytical process is provided on the manufacturer's website (HeartFlow, 2019).

Another example is IDx-DR, an autonomous system for diagnostic screening of patients with diabetes for diabetic retinopathy, a serious condition of retina that can lead to blindness, based on fundus images of the eye (IDx-DR, 2019). IDx-DR's diabetic retinopathy detection device received FDA clearance on April 11, 2018 *via* the De Novo (DEN180001) regulatory pathway (classification product code PIB, regulation number 21 CFR 886.1100, class II, 510(k) regulatory pathway; (FDA, 2018a)]). IDx-DR's software contains two types of ML algorithms: the first one for image processing, outputting a binary classification of whether the images are of sufficient quality to be properly analyzed by the diagnostic algorithm and the second one using multilayer convolutional neural networks and a multiscale feature bank detector, to distinguish between patients suffering from more than mild diabetic retinopathy (and should therefore be referred to a specialist) and patients who do not (and therefore should be re-screened in a year). This solution is autonomous, meaning that it does not technically require a clinician to make a medical decision. Finally, OsteoDetect is a software device used to help clinicians diagnose and locate wrist fractures. Similarly, as in the case of the IDx-DR, the tool first determines whether an X-ray image is eligible for processing, and if yes, it applies an ML-based algorithm to this set of images in order to establish the probability (confidence) of bone fracture. If the fracture is deemed present, another algorithm will also map the X-ray image, highlighting the location of the alleged fracture (conditional probability map). This device is intended as an adjunct tool, meaning that it is only a decision support, presenting to a physician both the unaltered and altered radiographic images for a final clinical decision.

AI-based diagnosis support can be considered as a classification method. Hence predictive power and Receiver Operator Characteristics Area Under the Curve (ROC AUC) of such algorithms are typically evaluated based on a “ground truth” established *via* majority vote of three board-certified specialists. The established sensitivity and specificity of the featured examples is around 90% (Danad et al., 2017; Driessen et al., 2019). IDx-DR achieved sensitivity of 0.87 and specificity of 0.91 (Abramoff et al., 2018). Heart Flow compared to alternative non-invasive tests such as Coronary computed tomography angiography (CCTA), single-photon emission computed tomography (SPECT), and stress echocardiography (SE) with ROC AUC 0.94 when evaluated in a prospective study with 208 patients and 504 vessels (sensitivity 0.90 and specificity of 0.86) (Driessen et al., 2019). In the case of OsteoDetect where the result is a conditional probability map, the accuracy of localization is established using a centroid method, analyzing how far the actual fracture is located from the area pointed out by the device.

Another parameter of interest is whether and by how much a device-made or machine-assisted tool exceeds the quality of prediction of non-specialist human graders, such as general practitioners. For example, OsteoDetect improves the ROC AUC by 0.049 (FDA, 2018c). HeartFlow Analysis improves ROC AUC by 0.11, 0.12, and 0.24 compared to CCTA, SE, and SPECT, respectively (Driessen et al., 2019).

This is typically established in observational studies (both retrospective and prospective) using several hundred to several thousand images. However, prospective clinical trials have also been conducted. One prospective clinical trial includes HeartFlow's Prospective Longitudinal Trial of FFR_CT_: Outcome and Resources Impact (PLATFORM NCT03310619) trial, which included 584 patients with new onset chest pain and intermediate likelihood of CAD (ACC, 2019, April 17). Another prospective clinical trial includes HeartFlow's Prospective Randomized Trial of the Optimal Evaluation of Cardiac Symptoms and Revascularization (PRECISE NCT03702244) trial, which included approximately 2,000 patients with stable typical or atypical symptoms suggesting possible CAD (Douglas et al., 2015; Douglas et al., 2016). The advantages of a prospective study is that it allows for safety follow-up and establishes whether the patient in whom invasive coronary angiography was deferred based on the results of Heart Flow analysis experiences adverse events within one year (Douglas et al., 2016).

The algorithms are typically incorporated in a client software installed on local computers at the point of care. Analysis can be made locally or be transferred *via* internet to an external server of the device provider for analysis. For example, HeartFlow^®^ contains an application that transfers images *via* Digital Imaging and Communications in Medicine (DICOM^®^) protocol to Amazon Web Services Cloud from a local computer or directly from a CT scanner. Whenever medical data are transferred, controls need to be implemented to assure data privacy and other aspects of computer security. User-experience studies can be conducted to determine an optimal way of interaction between the device and its operator. The systems operators at the point of care need to be trained. The training can be as minimal as 4 h in total, in the case of general practitioners who had never undergone ocular imagining training to use IDx-DR.

All of the devices previously described are regulatory-agency approved (Software as Medical Device [SaMD]) and currently marketed. All of them were developed with an intention to optimize healthcare, either by sparing the risk and cost associated with a diagnostic procedure (e.g., HeartFlow^®^) or by shifting the point of clinical decision making from specialist to primary/emergency care (e.g., IDx-DR and OsteoDetect), or by reducing the need for training among specialists (e.g., OsteoDetect).

### Case 3: Clinical Decision Support System

A promising field for the application of ML in medicine is choice of treatment. An example of this application is a clinical decision support software developed by Pacmed, a medical ML developer from the Netherlands. The application is built for the support of physicians in prescribing antibiotics for patients with urinary tract infections.

The guidelines in prescribing treatment choices, which are based on clinical studies such as randomized clinical trials (RCTs), are rather homogeneous (Lugtenberg et al., 2009). Due to the exclusion criteria for these studies concerning comedication, comorbidities, and risk minimization, a non-representative study population is included.

Using ML, a CDSS that generates personalized guidelines for every individual of the actual population was developed. To this end, the characteristics of over 200,000 patients, including age, gender, disease characteristics, comedication, comorbidities, diagnostic results, and the details of previous urinary tract infection episodes, were analyzed. By combining this with the type of antibiotics prescribed and whether this intervention was successful for every individual case, an ML algorithm was able to predict the chances for the successful elimination of the infection for the different treatment choices.

The development of such a CDSS knows numerous challenges. First, accumulating a sizable dataset *via* individual physician's practice is near impossible. Therefore, an aggregated dataset, collected by the Dutch healthcare research organization NIVEL, was used for the development. Collaboration with such a trusted third party also ensures the privacy of included patients due to the level of anonymization applied by the researchers aggregating the dataset.

Second, the successful implementation of a new CDSS in the healthcare system requires adequate validation and certification of the system. The developers are currently validating the system in an implementation pilot study where the system is tested in 120 practices. Based on the results of this pilot study, the external validity of the system will be assessed (the extent to which the model generalizes to the new unseen population). When this study is concluded positively, the developers can initiate the final step before market introduction, which is the application for CE-certification (applicable to the European Union [EU]) for medical devices.

The third challenge in this development are the limitations in the pilot study for integrating the new system in the existing software that the health care practictioner uses. Full integration in the existing software is often not feasible for a pilot study, which means that the developers have to account for the suboptimal user experience of the system. As such, the pilot study is to be used in parallel to the existing software requiring registration information in both systems. The results of the pilot study, in terms of usability, actual use, and impact, therefore, have to be corrected for the fact that the system that was used provided barriers for use that would not be present in the implementation of the actual system.

### Case 4: Precision Dosing

All prescribers recognize the feeling of frustration when drug therapy fails for their patients and the feeling of regret when prescriptions cause adverse drug reactions or intolerable adverse effects. These two outcomes occur ubiquitously in clinical medicine despite drugs being prescribed, in most cases, as directed by the manufacturer and/or clinical practice guidelines. Such adverse clinical outcomes are not trivial, costing around $US 42 billion globally per year (WHO, 2017). There have been renewed efforts to individualize drug therapy for patients since the Precision Medicine Initiative was announced by the Obama administration in 2015 (Terry, 2015). This partly involves CDSS to optimize therapeutic pathways and drug selection (see *Case 3*), but also recognition of the importance of drug dose as a determinant of clinical outcomes. Precision dosing has recently been defined as “dose selection by a prescriber for an individual patient at a given time” (Polasek et al., 2018). This definition covers initial dose selection following the decision to commence drug therapy, and review of dose based on the benefits/risks of ongoing treatment. Advanced quantitative approaches for precision dosing are available, and these are now collectively being described as “MIPD” (Wright et al., 2019).

Drug therapy outcomes are improved using MIPD for drugs with narrow therapeutic indices (TIs) in difficult-to-dose patients for whom the clinical stakes are high, such as anti-microbial use in patients who are hemodynamically unstable critically ill (Rawson et al., 2018). The most successful MIPD approach is the use of population pharmacokinetic/pharmacodynamic models (pop PK/PD) with Bayesian priors that are updated post-dose with plasma concentrations from therapeutic drug monitoring (TDM) to generate PK parameters for the drug in the individual patient, thus allowing Bayesian forecasting of the dose needed to obtain the target plasma concentration. A recent example highlighting the value of this approach to precision dosing is the RCT of paclitaxel-based chemotherapy in advanced non-small cell lung cancer comparing MIPD with traditional dosing based on body surface area (BSA). In this clinical study, which involved >300 patients, there were significantly lower rates of paclitaxel-induced toxicities (grade 4 neutropenia and > grade 2 neuropathy) in the MIPD arm compared to the BSA arm, without compromising efficacy (Zhang et al., 2019). In contrast to Bayesian forecasting, the use of AI/ML-derived algorithms as DSTs for precision dosing has received relatively little attention. By way of example, two cases from the literature are now presented where AL/ML was used to inform dose selection in clinical practice.

Several Artificial Neural Networks-based (ANN) software can be used for precision dosing. An “anemia control model” (ACM) was recently constructed and used to support the dosing of darbepoetin, an erythropoietin-stimulating agent (ESA), in patients with anemia and end-stage kidney disease who require hemodialysis (Barbieri et al., 2015; Barbieri et al., 2016). The clinical goal was to achieve stable hemoglobin (Hb) concentrations within a target Hb range, thus improving the symptoms of anemia such as shortness of breath, fatigue, and exercise intolerance. As the lifespan of erythrocytes is about three months, darbepoetin dose during these months is considered in the ACM simulations, together with sampling times, dialysis treatment, and inflammatory markers, iron studies, and other biochemistry results. The software simulates the effect of different ESA doses and determines the optimal dosing regimen to obtain the target Hb concentration. The software learns through the data encountered. The computational model is composed of layers of connected units called neurons, exchanging information through weighted connections. The ACM was initially built and retrospectively tested on data from >3,000 patients undergoing hemodialysis, showing that 90 to 93% of a test dataset had a percentage of error lower than 1 g/dl in Hb concentration (Barbieri et al., 2015). The ACM was then further tested by comparing a control phase of standard of care (n = 640) and an observational phase (n = 752), in which nephrologists were provided darbepoetin dose suggestions by the ACM (note that the nephrologists could accept or reject the ACM suggestions). The main findings of the study were: (1) the monthly darbepoetin doses were lower (0.62 µg/kg/month versus 0.46 µg/kg/month), (2) the percentage of patients with target hemoglobin were higher (70.6 to 82.3%), and (3) hemoglobin fluctuations were lower (intrapatient standard deviation [SD] from 0.95 to 0.83 g/d) in the observational part of the study compared to the control phase (Barbieri et al., 2016). Thus, AI/ML-based precision dosing of darbepoetin was superior in the management of anemia in patients with end-stage renal disease requiring dialysis, compared to experienced nephrologists.

A recent study published in Nature Medicine described the development and performance of another AI-supported model for precision dosing, this time using reinforcement learning (Komorowski et al., 2018). Reinforcement learning is a category of AI in which a virtual agent learns from trial-and-error approach using an optimized set of rules—a policy that maximizes an expected return (Bennett and Hauser, 2013). An “Artificial Intelligence Clinician (AI Clinician)” was used to guide the selection of vasopressor doses and intravenous (IV) fluids for patients with sepsis in the intensive care unit (ICU). The AI model was highly multifactorial, including a large number of clinical and biochemistry parameters. It was developed and implemented based on admissions in ICU databases in the US, with 80% of the data used for development and 20% for validation. In the validation cohort, in-ICU, in-hospital, and 90-day mortality were significantly lower when prescribers followed the AI Clinician's suggestions on vasopressor doses and IV fluid administration. These data suggest that AI/ML-based precision dosing exceeds by many-fold the lifetime experience of human clinicians in the improvement of clinical outcomes in the ICU.

As an epilogue to these two examples, an important application of AI/ML in the field of precision dosing is to improve the definition of precision dosing targets e.g., plasma drug concentrations, biomarkers of pharmacodynamic effects, etc. This is currently done by understanding PK/PD relationships using various modeling approaches, predominantly during drug development, such that the “sweet spot” for the dose that generates the best balance of benefit/risk for a particular type of patient is determined. The data are then used in an argument to support a limited number of dose options when seeking marketing approval. Since AI/ML approaches can accommodate very large datasets, precision dosing targets based on real world evidence of clinical outcomes are likely to be superior to those determined from limited datasets in drug development. Furthermore, it is envisaged that AI/ML will be increasingly important to support Bayesian forecasting for MIPD based on traditional pop PK/PD models and TDM e.g., DoseMe^®^, InsightRx^®^, and TDMx^®^ software. Various AL/ML approaches could be incorporated into software to enable learning and model optimization during clinical use.

## Regulatory Requirements for AI/ML Solutions

When AI/ML is applied to the “treatment, diagnosis, cure, mitigation, or prevention of disease” the software is referred to as SaMD in the US (FDA, 2019a; IMDRF, 2013), and referred to as Medical Device Software (MDSW) in the EU (EU Commission, 2019). Regulatory requirements depend on the level of risk associated with the use of the device. Generally, medical devices comprising algorithms are the highest risk class if their use involves surgical invasion, surgical implantation, or administration of certain drugs to patients who are medically compromised. Most devices using a software algorithm are moderate risk, and a few are considered low risk.

The regulation of AI/ML systems presents specific challenges. The level of self-learning, whether it is supervised learning on curated data, reinforcement learning, or possibly unsupervised learning, impacts the challenges present for the manufacturer of the software. This would include software safety and the definition of the intended use, keeping the initial clinical evaluation of the software valid. A controlled design process that uses AI/ML in the development and collection of data during clinical use, which could be used in the development of the next version of the software, is a way of keeping control. The regulation of software with continuous learning represents a task for the future.

## Discussion

A recent review by McKinsey and company (Batra et al., 2018) outline the point of consideration and potential benefits/risks of application of AL and especially for ML and DL technologies in various industries. Nine layers are identified as the type of ML/DL technology where companies from healthcare, pharmaceutical, and medical devices sectors can provide a particular single/double and multiple layers. Definition and examples of each of layer such as (1) accelerator (hardware), (2) head node (hardware), (3) interface, (4) framework (platform), (5) algorithm (platform), (6) architecture (platform), (7) methods (training), (8) data types (training), and (9) solution + use case (services). Of the AI demand and opportunity in various industries, healthcare has been identified to consist of a global market size of $5 to $10 trillion USD, with over 50 use cases, over $1.0 billion USD start-up equity, and 15 to 20% economic impact (where economic impact is the sum of the value related to all use cases divided by the global industry size). By comparison, pharmaceutical and medical products encompass <$5 trillion USD, 10 to 30 use case, <$0.5 billion USD for business start-up, but surprisingly providing >20% average AI economic impact (where the economic impact is defined as the sum of the value related to all use cases divided by the global industry size) (Batra et al., 2018).

### Potential and Opportunities of AI/ML Solutions

ML, embedded in medical devices or as a standalone software, has a potential to transform clinical practice and enhance patient outcomes while cutting down the healthcare costs (Hlatky et al., 2013; Padhy et al., 2019). This transformative potential will be realized when ML algorithms: (1) replace a physician in making a clinical diagnosis, (2) help resolve a clinical question in the primary rather than specialist care setting, (3) make it possible to effectively monitor a patient at home instead of at a hospital, and (4) make an accurate judgment about the type of treatment and dose of a drug.

Above we provided examples of marketed AI/ML-based solutions across clinical continuum that hold to this promise. These are either autonomous, meaning that they do not technically require a physician to make a medical decision (t:slim X2 insulin pump, IDx-DR) or adjunct, meaning that they support decision making by providing additional evidence or recommendation, for consideration of a physician (all other presented examples). The support from AL/ML algorithm to continue learning and evaluating the data will shorten the time and lower the cost to provide health solution (e.g., diagnosis of a disease), improve accuracy from human-alone interpretation/evaluation, accelerate the solution for patients as compared to traditional approach, and prevent disease worsening or save life. Such solutions can bring measurable savings. For example, Skin Analytics^®^ software for the identification of potentially cancerous skin lesions, reduced onwards referrals from primary to specialist care by around 50% (Phillips et al., 2019). A National Health Service (NHS) study conducted in Bristol in 2011, found that reducing the onward referral around this level could save £43,000 per 100,000 population (NHS, 2019).

Some of the benefits of the AI/ML solution include knowledge continuum integration with fast growing global databases that collate continuous collection of patient data as part of the AI/ML solutions and during the process of system learning from emerging data. AI/ML might also provide recommendation to potential imperfection of current data collection methods, which allows gap analysis of current sample collection approach. In parallel, due to the big data element, uncountable combinations of patient demographic to disease pathology and progression will advance the understanding of variability in identifying a certain disease and the variability in the response of the disease to the designated treatments.

### Challenges for AI/ML Solutions

Even though the number of promising AI/ML-based technologies is increasing, still, only a few have been implemented widely at the point of care. Challenges occur primarily at the stage of access to data for algorithm development, algorithm validation and implementation at the point of care.

#### Data Availability and Quality

One of common obstacles in development of ML-based solutions based on retrospective data is access to suitable, large data for model training and validation. While the strengths of ML are particularly visible when algorithms are developed and validated on large databases, such as Electronic Medical Record (EMR) or clinical registries, access to such databases is limited by data protection policies and high costs. Currently existing European data protection laws and data governance models, for which the General Data Protection Regulation (GDPR) sets the minimal standard, require that a specific consent be given by a patient for each use of their data. Many data sources were consented only for the original, specific purpose of their collection. Absence of a broad consent for future data repurposing can therefore limit usability of high quality clinical data. Even when consent for data repurposing is granted, another data protection and privacy challenge is difficulty in pseudo-anonymization of images, which are always marked with a patient identifier, and therefore, require additional processing before they can be reused, which increased the cost of data access and prolongs the timelines.

Once access to such data is granted, a quality challenge remains, as clinical databases were typically not designed with development of an ML-based algorithm and then software in mind. As an effect, data incompleteness or a discrepancy between clinical practice and the information captured in the database can occur. For example, information about non-adherence is typically missing, but could result in inefficacy of the prescribed therapy. Objective clinical outcome data can also be missing, in which case a surrogate for clinical outcome could be considered. For instance, when a patient with UTI does not return to the physician, it means that the infection was likely resolved. However, it cannot be precluded that the patient migrated or died. Requested information may not always be available to the reporter, or the description of the data item may not be fully clear for the reporter. The provided information could be a random number or a default option and may not be accurate.

It can be of interest to pool data across centers/systems/regions, which could result in challenges regarding data integration and harmonization. Finally, crucial clinical information might have been captured in free text. Data mining techniques could then be considered, but these may not be sufficiently accurate. Manual data processing is a resource intensive alternative, which would require review of clinical reports, covered by a confidentiality arrangement.

#### External Validation and Validity

ML algorithms per design achieve a high level of internal validity that might be spurious (Pafitis et al., 2019) therefore, their validation is necessary, ideally a cross-validation at the stage of development (using a split sample) and external validation before regulatory submission. To obtain trustworthy estimates, sensitivity and specificity of ML algorithms should be tested in a real-world setting of usual care. Factors such as the type of device used for imaging, level of training of operators, selection of participants to the study can otherwise elevate sensitivity and specificity obtained from an RCT. In case of analysis of images, interoperability plays a role and differences between images generated using different devices on the market need to be reconciled by calibrating the ML algorithm to each image-capturing device.

These technical aspects are, however, just one facet of validity. Another one is that despite the development of better drugs, many drugs still do not work in real-world patients, either because the drug is being used differently or the patient population is more selective than those in the clinical trials. Therefore, while selecting databases for the development of ML algorithms, it is important to ensure that the test data will be representative for the target patient group. Heterogeneity of datasets used for development and validation can be a threat to the validation but can be addressed through a thorough statistical design of the study, e.g., through bridging.

#### Implementation at the Point of Care

As a prerequisite for successful implementation at the point of care, it is important that the algorithm is readily accessible and easy to use. The software would ideally be integrated with the EMR and should be available to the end-user as part of the standard workflow at the point of care. Particularly in support of a multidisciplinary decision-making process, integration with other clinical decision support software can support effective implementation.

The output of the algorithm should foster optimization of treatment or diagnosis. The output should therefore be aligned with daily clinical practice. As an example, if the software indicates that the optimal dose would be 1.5 tablet while the tablet is not divisible, the algorithm will not successfully support dose selection.

From an end-user perspective, ML algorithm-based information should be appropriately interpreted. A typical pitfall can be, that the results are considered as irrefutable. However, a clinical case might ‘fall out of data', i.e. might not be represented by the data that were used to develop the ML algorithm. Another pitfall is, that the ML algorithm may not be based on causal relationships, while clinicians are used to causalities.

Data privacy plays a critical role not only at the development stage, but also after deployment of the ML solution at the clinic, especially if prospectively collected patient data need to be transferred to a centralized server for analysis. Providers of such solutions need to assure an appropriate level of data protection and computer security.

#### Ethics of the AI/ML-Based Solutions

AI/ML based solutions are prone to all types of biases in classical computer systems—the preexisting social biases influencing the way a software is designed, the biases emerging from how software is being used, and purely technical biases (Friedman and Nissenbaum, 1996). In this context, the critical ethical question is whether the algorithm can be trusted—and not only whether it produces technically “true” results but also if it acts in the best interest of patients in the way that a physician does Char et al., 2018; Gubbi et al., 2019). One prominent problem with algorithm's fairness is the potential for discrimination of certain groups of patients, particularly if such patients were not properly represented in data used for the algorithm development. The problem, though, is that ML-based algorithms with their “black-box” character are inscrutable by design which makes it very difficult to discover their malfunctioning resulting in unfairness (Mittelstadt et al., 2016). EU addressed this problematic in the GDPR, where the concepts of “right to explanation” and “Explainable AI” are introduced. The idea behind them is that an individual who is affected by a result produced from an AI/ML algorithm has the right to know the reasons behind the result (i.e., gets at how AI arrives at the decisions it does; how the decision was arrived at). The process and rationale for the outcome should therefore be disclosed as an explanation to individuals or subject experts. Another regulation contained in the GDPR is Article 22, which states that “individuals have a right not to be subject to a decision affecting them in a significant way if this decision is made by an automated system without human input” which could be interpreted as a rationale against use of autonomous ML-based system not requiring clinician input on the European market. Finally, it is also possible that an AI/ML algorithm is intentionally designed in a biased way, e.g. to favor diagnoses which are more profitable in certain healthcare systems (Char et al., 2018) or to recommend treatments produced by a particular company. Last but not least, a question needs to be asked about the role, responsibility and accountability of a physician in the emerging clinical practice where automatization and ML-based decisions will play an increasing role. Real-life examples are still scarce but the literature points to a number of anticipated problems. These range from dispersed or even removed responsibility in case a ML-based solution—created, approved and applied by a long and often opaque chain of actors—malfunctions (Mittelstadt et al., 2016), up to a blunt speculation whether AI superior performance in quickly and objectively analyzing complex and large volume evidence will eventually render physicians obsolete (Goldhahn et al., 2018).

### Regulatory Requirements

Regulatory oversight of AI/ML appears focused on ensuring safety through a system of verifications. Some concepts (Japan) aim at verifying the software itself (Banks, 2019), while others (US) are aimed at verifying the operational excellence of the business entity that developed the software.

Regulatory requirements in the US, Europe and China depend on the level of risk associated with the use of the device in medical practice, and can be classified into administrative (manufacturing and quality control), software-related (design, specification, hazard analysis, architecture, traceability, software risk analysis, cybersecurity, etc.), clinical evidence (including patient perspectives in some cases), non-clinical evidence (dosing validation and biocompatibility/toxicology) and other, such as e.g. benefit-to-risk determination, risk assessment and mitigation.

Generally, there is an alignment between the US and Europe. The differences include the addition of a Clinical Evaluation Report and the potential for additional clinical evaluation required for class IIa and class IIb devices in Europe. China additionally requires that the clinical evidence is applicable to the Chinese population, including clinical characteristics related to race, local epidemiology, and diagnostic and treatment practices, among other requirements (NMPA, 2019). This might include sampling pharmacokinetic data from Chinese patients in China or conducting a separate clinical study in China. A specific characteristic of the Chinese regulatory requirements is that analysis of the wet samples and clinical trials in general needs to be performed by a third-party central laboratory located in China (NMPA, 2019).

## Future Perspectives

Despite the popularity of AI/ML/DL technologies (Fleming, 2018) and the positive press of many applications to healthcare and the pharmaceutical and medical devices industry, a large proportion of researchers have yet to uncover the full capabilities of such advanced technologies to drug research and development. The future moving forward, would require AI to be embedded to a part of the educational system, especially at the graduate level. The recent (Madabushi et al., 2019) demonstrations, adoption, and acceptance of the use of model-informed drug development/pharmacometrics (PMX) to support drug discovery and development can be seen from both pharmaceutical industries and regulatory authorities.

There are many opportunities to combine the strengths of PMX and ML (Chaturvedula et al., 2019). PMX models are constructed with differential equations and can incorporate (semi-) mechanistic knowledge that are based on biological and pharmacological principles. PMX models may become quite complex (e.g., physiologically based pharmacokinetic model and quantitative system pharmacology models (Danhof, 2016)]) and covariate selection can be time-consuming. On the other hand, ML is a data-driven analysis approach without mechanistic components. As such, ML-based approaches are capable of analyzing large datasets almost in real time.

Data-driven ML scientists efficiently “train models” utilizing large datasets, whereas pharmacometricians “develop (semi-)mechanistic models” leveraging scientific knowledge. These different strengths give us the opportunity to combine PMX and ML methods. ML-based approaches can facilitate development of PMX computer models by streamlining screening and selection of covariates, while mechanistic PMX components can be incorporated in ML-based algorithms to enhance real-time clinical decision support (Hutchinson et al., 2018). Another application of AI in PMX is in the area of oncology (Houy and Le Grand, 2018). A Monte-Carlo tree search algorithm (from a class of AI) was embedded as part the protocol design to optimize the temozolmide dose using emerging exposure, toxicity, and efficacy data. This approach is superior to the traditional maximum tolerated dose, as outlined in the temozolmide example, and able to handle population complexity and variability from oncology patient's data. Another example seen in oncology is from FDA's publication on application of ML for time-to-event analysis in oncology (Gong et al., 2018).

## Conclusions

ML can deliver effective algorithms supporting practitioners in their daily work, with functions spanning from clinical monitoring through model-based precision dosing. While most of them will only provide recommendations to be reviewed and considered by physicians, others are designed to work autonomously and achieve higher accuracy than the physicians themselves.

Despite an increasing number of promising AI/ML-based technologies, few have been implemented widely at the point of care. The need for external validation, data exchange and privacy, and implementation logistics remain the main obstacles. Regulatory requirements depend on the level of risk associated with the use of the device in medical practice, and the regulatory practice of ML-based medical devices is currently being developed. We expect a rise in the use of ML-based solutions on the healthcare market, including in the precision dosing segment, where ML will become a part of the model-informed precision dosing.

## Author Contributions

ZA, LH, NH, EG, and AZ contributed to conception of the study. All authors wrote sections of the manuscript. All authors contributed to manuscript revision, read and approved the submitted version.

## Conflict of Interest

Author JZ was employed by the company PacMed.

The remaining authors were employed by Certara and declare that the research was conducted in the absence of any commercial or financial relationships that could be construed as a potential conflict of interest.
